# Bearing Aluminum-Based Alloys: Microstructure, Mechanical Characterizations, and Experiment-Based Modeling Approach

**DOI:** 10.3390/ma15238394

**Published:** 2022-11-25

**Authors:** Ahmed O. Mosleh, Elena G. Kotova, Anton D. Kotov, Iosif S. Gershman, Alexander E. Mironov

**Affiliations:** 1 Mechanical Engineering Department, Faculty of Engineering at Shoubra, Benha University, Cairo 11629, Egypt; 2 Department of Scientific Research Programs, Grants and Projects, Railway Research Institute JSC “VNIIZHT”, 3rd Mytischinskaya St. 10, 107996 Moscow, Russia; 3 Physical Metallurgy of Non-Ferrous Metals, National University of Science and Technology “MISiS”, Leninsky Prospekt, 4, 119049 Moscow, Russia; 4 Joint Stock Company Railway Research Institute, Moscow State Technological University “Stankin” (MSTU “STANKIN”), 127055 Moscow, Russia

**Keywords:** aluminum alloys, anti-friction materials, material design, mechanical properties, microstructure, neural network

## Abstract

Due to the engine’s start/stop system and a sudden increase in speed or load, the development of alloys suitable for engine bearings requires excellent tribological properties and high mechanical properties. Including additional elements in the Al-rich matrix of these anti-friction alloys should strengthen their tribological properties. The novelty of this work is in constructing a suitable artificial neural network (ANN) architecture for highly accurate modeling and prediction of the mechanical properties of the bearing aluminum-based alloys and thus optimizing the chemical composition for high mechanical properties. In addition, the study points out the impact of soft and more solid phases on the mechanical properties of these alloys. For this purpose, a huge number of alloys (198 alloys) with different chemical compositions combined from Sn, Pb, Cu, Mg, Zn, Si, Ni, Bi, Ti, Mn, Fe, and Al) were cast, annealed, and tested for determining their mechanical properties. The annealed sample microstructure analysis revealed the formation of soft structural inclusions (Sn-rich, Sn-Pb, and Pb-Sn phases) and solid phase inclusions (strengthened phase, Al_2_Cu). The mechanical properties of ultimate tensile strength (σ_u_), Brinell hardness (HB), and elongation to failure (δ) were used as control responses for constructing the ANN network. The constructed network was optimized by attempting different network architecture designs to reach minimal errors. Besides the excellent tribological characteristics of the designed set of alloys, soft inclusions based on Sn and Pb and solid-phase Cu inclusions fulfilled the necessary level of mechanical properties for anti-friction alloys; the maximum mechanical properties reached were: σ_u_ = 197 ± 7 MPa, HB = 77 ± 4, and δ = 20.3 ± 1.0%. The optimal ANN architecture with the lowest errors (correlation coefficient (R) = 0.94, root mean square error (RMSE) = 3.5, and average actual relative error (AARE) = 1.0%) had two hidden layers with 20 neurons. The model was validated by additional experiments, and the characteristics of the new alloys were accurately predicted with a low level of errors: R ≥ 0.97, RMSE = 1–2.65, and AARE ˂ 10%.

## 1. Introduction

**Figure 1:**
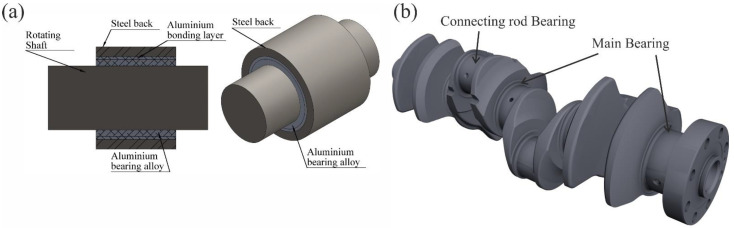
(**a**) Typical construction of metal bearing using Al-based alloys, (**b**) the bearing positions on the crankshaft.

Due to their high mechanical and good tribological properties, lead, and tin-lead bronzes remain the most frequently used material for journal bearings. In addition, friction forces and their effects consume approximately 23% of the world’s energy today [1,2,3]. Due to the substantial cost savings associated with bearing production and subsequent maintenance, the transition from bronze to aluminum-based alloys has become a driver for the engine-building industry’s development [4]. Compared with copper-based alloys, aluminum-based alloys are three times lighter, 2.5–2.7 times cheaper, easier to process and manufacture, and require less energy to cast than copper by 15–20%. Aluminum, unlike bronze, does not act as a surfactant on steel and does not attack it through the Rehbinder effect [5]. As a result, further chemical-thermal treatment of steel shafts can be avoided when using aluminum-based anti-friction bearings, and thus anti-friction alloys have gained significant interest [6,7,8,9,10]. Figure 1 demonstrates the construction of Al-metal bearing shells, as well as their constructions and positions on the crankshaft, as one example of the various uses of metal bearings. The aluminum anti-friction bearing shell is assembled on the steel back substrate using the aluminum bonding layer (Figure 1a). The anti-friction bearing alloys were developed to fulfill various requirements such as good mechanical properties, high corrosion and wear resistance, excellent thermal conductivity, and lubricity, and they should be cost-effective materials [11,12,13]. Figure 1b shows the positions of the bearing on the crankshaft and the bearing construction used in the modern high-speed internal combustion engine [14]. Any bearing material must have high compressive strength and fatigue resistance to prevent permanent deformation and the formation of permanent deformation and forming surface fatigue cracks.

The proper selection of bearing materials is crucial for securing the operation of friction units and avoiding failure. Materials of monometallic bearings for capacity expansion and toughness operations should exhibit good mechanical properties with good anti-friction properties [15,16,17,18,19,20]. Lead and tin are considered the most common alloying elements in aluminum anti-friction alloys [21,22,23,24]. Because of its excellent tribological performance, the Al-20%Sn-1%Cu alloy is regarded as a fundamental alloy for designing novel anti-friction materials. However, due to its inadequate mechanical characteristics, it can only be used in bimetallic bearings. As previously demonstrated [1,11,25,26,27], multicomponent Al-based alloys display excellent tribological characteristics due to the synergetic interaction between alloying elements of the multicomponent alloys. In addition, Sn, Pb, and Bi are used as components that create soft-phase components. Silicon (Si), like iron (Fe), is always present as an impurity with aluminum. In bearing alloys, magnesium (Mg) is added in small amounts (up to 2%) to increase the strength of these alloys [11]. Mg can be problematic in the presence of tin (Sn) since it forms a chemical compound Mg_2_Sn with Sn. However, with complex multicomponent alloying, the formation of the Mg_2_Sn phase can be suppressed or completely absent. In this case, Si addition results in the formation of the Mg2Si phase and the Guinier–Preston zone, which contribute to the alloy’s hardening during the aging process. Copper (Cu) increases the modulus of elasticity of Al-based alloys, and is considered one of the main alloying additives that sharply harden aluminum alloys. Natural aging is capable of hardening Al-Cu alloys. Fe has a detrimental effect on the aging process of Al alloys, while the addition of small Mg additives (0.02–0.03%) restores the aging ability of these alloys. Zinc (Zn) is an ineffective solution hardener, making its use as the only alloying element impractical. Nevertheless, when it is alloyed with other additives together, particularly with Mg and Cu, it significantly influences the properties of the produced alloys. Adding Mn to Al-based alloys strengthens them by the formation of a solid solution. This solution decomposes during subsequent technological heating at elevated temperatures, resulting in the formation of secondary aluminides, thereby hardening the alloys. In addition to hardening, transition element additives, such as Ti, often improve the workability of alloys by refining the grain. The addition of a small amount of Ni significantly increases the volume fraction of the eutectic and improves the casting properties. Alloying the strontium (Sr) to Al-Si-based alloys changes the Si morphology to fibrous structure from needle shape, resulting in a substantial improvement in the wear resistance of these alloys [28].

Several researchers have chosen computational analysis approaches, such as Finite element analysis [29] and ANN [30,31,32,33,34], for solving many engineering problems to avoid high-cost experimental methods that would need a lot of time, effort, raw materials, and expense. The prediction of the mechanical properties of the anti-friction aluminum alloys with a large number of alloying elements prior to their production is too complex. In order to resolve this problem, a large number of alloys are required to construct an accurate predictive model. ANN is the most popular modeling and predictive approach for simulating and predicting material properties. Additionally, it is an ML technique that originated and progressed from mimicking the human brain [30,31,32]. In recent years, ANN has provided fundamentally novel and different materials processing and modeling control approaches compared to numerical or statistical methods [33,34]. One of the primary advantages of ANN over constitutive analysis is that there is no need to postulate a mathematical model prior to employing neural networks. ANN learns from recognized trends and examples in a series of output and input values without any prior assumptions about the nature of the output and input values. Furthermore, the ANN model does not have any physical understanding of deformation and restoration requirements. Because of its ability to retain data in memory and its precise ability to deal with discrete data, ANN is highly recommended for modeling adoption. It also has a significant capacity to adapt the configuration of the old network to match the current experimental results [35]. ANN is well suited to estimating flow stress from available experimental data due to its inherent high parallelism.

Input, one/more hidden, and output layers make up a typical network linked by processing units known as neurons. All of the network’s neurons are connected together by weights. The output layer distributes the results to operators, and the input layer collects data from the outside. The hidden layer with a fixed number of neurons aims to avoid the complication of non-linear problems. The ANN model has the following steps: gather experimental results, define output/input variables; pre-process and analyze the gathered data; ANN training and monitoring; evaluate its performance. The main objective is to reduce the errors between the measured experimental targets and the ANN outputs. Backpropagation (BP) is a powerful machine learning approach for multi-layer expectations that involves adjusting weights and biases using a gradient descent strategy to reduce error for a specific training sequence [36,37]. 

Though the ANN is widely and effectively used in modeling the behavior of materials, from the literature, there is a huge knowledge gap in using the ANN for predicting the mechanical properties of anti-friction aluminum-based alloys. In this context, this work aims to: (1) Provide an accurate ANN architecture for predicting the mechanical properties of the anti-friction aluminum-based alloys (alloying elements: Sn, Pb, Cu, Mg, Zn, Si, Ni, Bi, Ti, Mn, and Fe) without performing extra experiments, thereby saving the time, effort and materials, and reducing the overall cost. (2) Understanding the impact of soft phases and the solid strengthened phases on the mechanical properties of these alloys. The soft inclusion elements provide the desired tribological properties, while the hard inclusion elements provide the required level of mechanical properties. It should be noted that this work is part of a more significant effort to replace bronze-based bearing materials with aluminum-based anti-friction alloys [26,27,38]. Consequently, the additional elements were selected considering the aim of this series of work, achieving good tribological properties with an acceptable level of mechanical properties without softening and failure. The ANN for modeling and prediction of the mechanical properties of such huge alloys is a feasible study for time and material saving, and to our knowledge, such a study has been performed here for the first time. 

## 2. Materials and Methods

### 2.1. Samples Preparation

**Figure 2:**
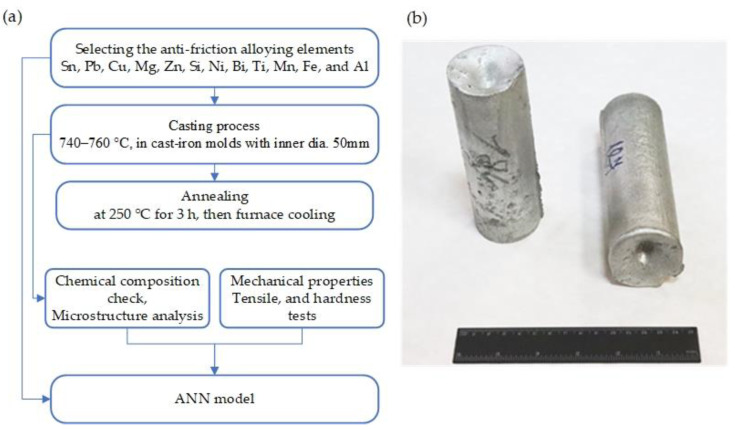
(**a**) Schematic flow chart of this work. (**b**) Image of the as-casted ingots of the investigated alloys poured in the iron mold.

Figure 2a depicts the schematic flow chart of this work, including the preparation and casting of the proposed alloy, the characterization step (microstructure and mechanical characterization), and the creation of the ANN model. In the current study, 198 Al-based alloys containing Sn, Pb, Cu, Mg, Zn, Si, Ni, Bi, Ti, Mn, and Fe elements were investigated. The content of the elements varies within the limits tabulated in Table 1. Pure Al (99.95), Sn (99.90), Pb (99.985), Bi (97), and Zn (97.5) were used for sample casting (all numbers are in wt%). Master alloys of Al-20Cu, Al-15Ni, Al-10Cu-10Ni, Al-10Si, and Al-12Mg were used to incorporate Cu, Ni, Si, and Mg elements. The master alloys were initially prepared from Al with mono metal Cu (99.90), Ni (99.7), Si (98.8), and Mg (99.95). According to the Russian standard, GOST (11069, 860-75, 3778-98, 10928-90, 3640-94, 859-2014, 849-2018, 2169-69, and 804-93), the purity of the alloying elements and the amount of the elements in master alloys were selected [39], as well as the binary phase diagram of each master alloy. Ti was added into the melted alloys in the form of salts hexafluorotitanic and hexachloroethane as part of the degasser-modifier “Zernolit-2” [40,41]. 

The melting processes of the investigated alloys were carried out in a high-frequency induction furnace using a graphite-fireclay crucible with a weight capacity of 15 kg. In order to ensure a good homogenization and distribution of elements, the melted alloys were maintained at 800–840 °C for 40 min. In addition, the melt was automatically mixed by eddy currents. First, aluminum was melted and held at a temperature range of 800–810 °C, then Al-20Cu, Al-15Ni, Al-10Cu-10Ni, Al-10Si, and Al-12Mg master alloys were added to the melt according to the composition of the desired experimental alloys. Prior to the pouring process, the corresponding weights of low-melting elements (Zn, Sn, Pb, Bi) were added last. Degassing and modifying of the investigated alloys were carried out using an intermediate crucible. Finally, the melt was poured at a temperature range of 740–760 °C into cast-iron molds with a diameter of 50 mm pre-heated to 200 °C. Figure 2b shows the casted ingots. The total duration of the pouring process, including the time required to leave the furnace and pour the entire quantity, was limited to between 150 and 180 s. During the casting process, the temperature was controlled by a chromel–alumel thermocouple. The ingots were subjected to a heat treatment that included annealing in a Nabertherm N 30/65A (Nabertherm GmbH, Lilienthal, Germany) electrical-resistance furnace at 250 °C for 3 h to stabilize the microstructure, followed by cooling with the furnace at room temperature [42].

### 2.2. Chemical Analysis

The casted alloys’ chemical compositions were measured using the spectral technique on Spectrolab-S installation (SPECTRO Analytical Instruments GmbH, Kleve, Germany). For this purpose, 30 × 30 × 20 mm samples were cut from the casted ingots. Before the chemical composition detection, the longest sides of the investigated samples were well-ground and polished. For precise measurements, the chemical composition was detected and recorded for five points on the longest sides of the samples.

### 2.3. Microstructure Analysis

For metallographic analysis, including grains morphology and study of formed phases, samples with different compositions (Table 2) were sectioned from annealed ingots, mechanically ground using various grades of SiC grinding papers ranging from 600 to 4000, and polished with emery cloth in a colloidal silica suspension, containing 20% water solution. Scanning electron microscopy (SEM), Tescan-VEGA3 (Tescan Brno s.r.o., Kohoutovice, Czech Republic) with an X-MAX80 (Oxford Instruments plc, Abingdon, UK) energy dispersive spectrometry (EDS) system was used for microstructure analysis, chemical analysis of alloy composition and phase microanalysis.

### 2.4. Mechanical Characterizations

**Figure 3:**
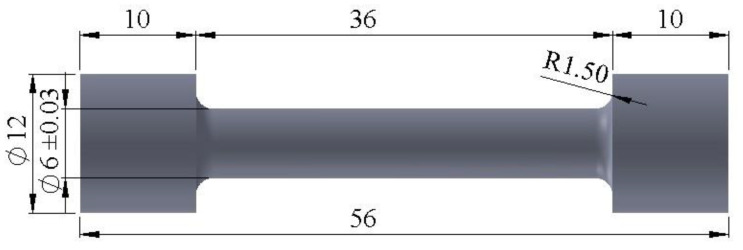
Dimensions of the tensile test specimen; all dimensions are in mm.

The tensile tests were conducted via a Zwick Z250 tensile testing machine (Zwick Roell Group, Ulm, Germany) using a cylindrical sample, with dimensions as shown in Figure 3, according to the ASTM E8 standard. Three tensile samples were tested for each alloy, and the mean of the three results was utilized to calculate the reported value. The tensile tests were performed at a crosshead speed of 5 mm/min. Each alloy’s tensile characteristics, such as tensile strength (σ_u_, MPa), and elongation (δ, %), were determined via the tensile test. HB of the investigated alloys was carried out by the Universal Hardness Testing Machine KB 3000 (Germany, KB Prüftechnik GmbH, Hochdorf-Assenheim). According to the ASTM E10 standard, the test conditions were ball indenter diameter = 2.5 mm, testing load = 612.9 N, and holding time = 30 s.

### 2.5. Mechanical Properties Modeling

The experimental data of tensile and HB were utilized to construct an ANN predictive model. A comparison study between one, two, and three hidden layers of network architecture was illustrated based on the prediction accuracy of each one. Statistical indices shown in Equations (1)–(3) were employed to assess the effectiveness of constructed networks. It is noted that the approach value of R is the unity while the approach values of *RMSE* and *RMSE* are both zero.

(1) $$R=\frac{\sum_{i=1}^{N}\left(W_{i}-\bar{W}\right)\left(Z_{i}-\bar{Z}\right)}{\sqrt{\sum_{i=1}^{N}{\left(W_{i}-\bar{W}\right)}^{2}\sum_{i=1}^{N}{\left(Z_{i}-\bar{Z}\right)}^{2}}}\;\;$$

(2) $$RMSE=\sqrt{\frac{1}{N}\overset{N}{\underset{i=1}{\sum }}{\left(W_{i}-Z_{i}\right)}^{2}}\;\;\;\;$$

(3) $$AARE=\frac{1}{N}\overset{N}{\underset{i=1}{\sum }}\left|\frac{W_{i}-\;Z_{i}}{W_{i}}\right|\;\;\;\;\;$$

where

- *W_i_*/*Z_i_*—experimental/predicted values,
- $\bar{W}/\bar{Z}$—average of the experimental/predicted values,
- *N* is the number of the casted alloys.

## 3. Results

### 3.1. Microstructure Analysis

**Figure 4:**
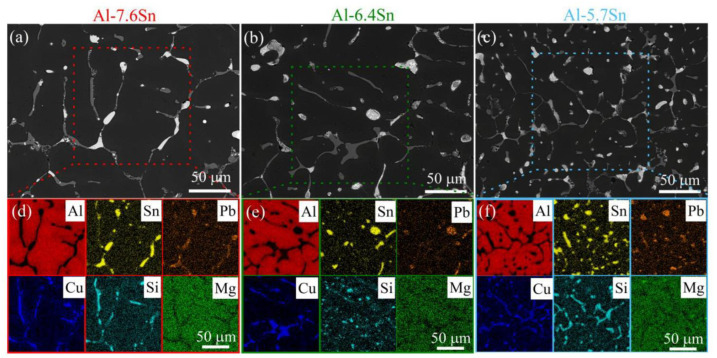
The SEM-BSE microstructures (**a**–**c**) and corresponding EDS-maps (**d**–**f**) of the investigated alloys: (**a**,**d**) Al-7.6Sn, (**b**,**e**) Al-6.4Sn and (**c**,**f**) Al-5.7Sn.

**Figure 5:**
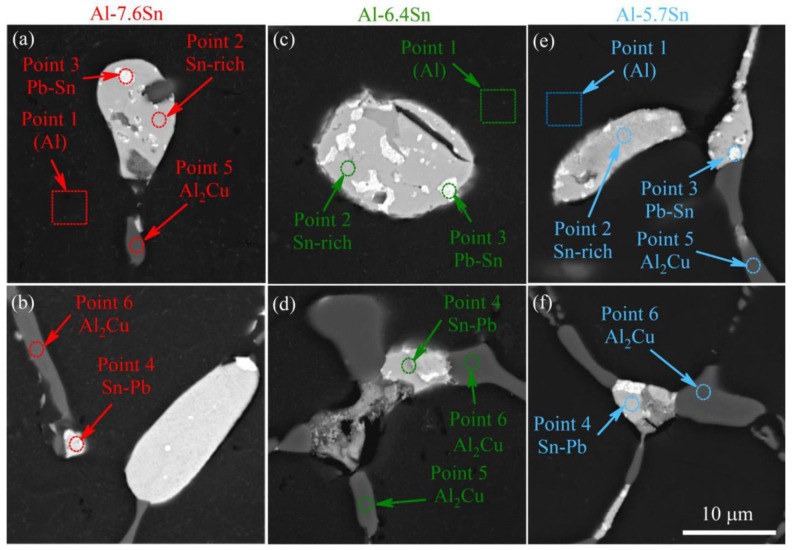
The BSE-SEM microstructures of the (**a**,**b**) Al-7.6Sn, (**c**,**d**) of the Al-6.4Sn, and (**e**,**f**) of the Al-5.7Sn alloys. The EDS spectra and chemical compositions of the analyzed points are presented in Figures S1–S3 in Supplementary data.

**Figure 6:**
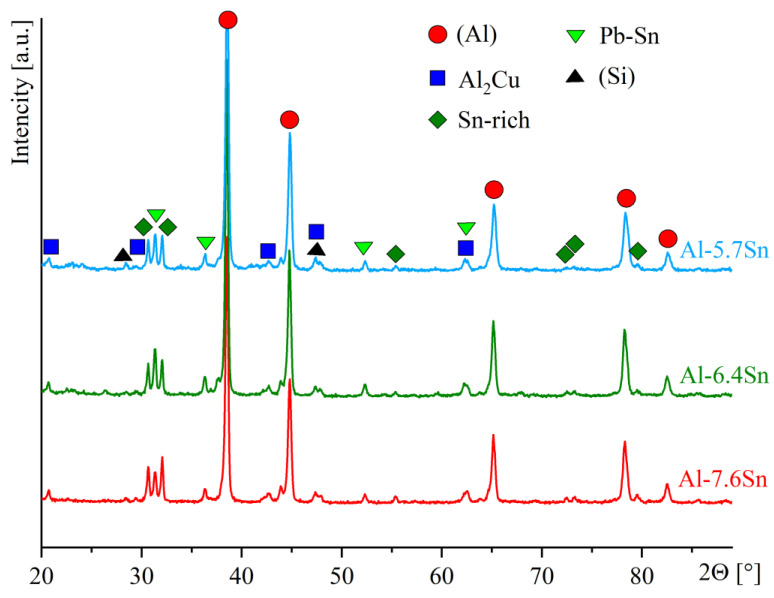
XRD patterns for the investigated alloys.

Figure 4 shows the SEM microstructures (Figure 4a–c) and corresponding element distribution maps (Figure 4d–f) of the selected alloys, Al-7.6Sn (Figure 4a,d), Al-6.4Sn (Figure 4b,e) and Al-5.7Sn (Figure 4c,f) after heat treatment at 250 °C for 3 h and cooled in the furnace to room temperature. The Al-7.6Sn exhibits a dendritic cell structure with a size ranging from 10–110 μm (Figure 4a). In addition to the aluminum solid solution (Al), the elements distribution maps (Figure 4d) reveal the presence of soft structural inclusions of Sn-and Pb-rich phases (Sn-rich, Sn-Pb, and Pb-Sn phases), as well as the solid phase inclusions of Al_2_Cu (*θ*) phase, as confirmed by [23,43,44,45]. Due to the close atomic numbers of Al and Si, the backscattered electrons (BSE) SEM images do not reveal the presence of a small amount of the (Si) phase in the microstructure. However, the Si distribution map demonstrates its presence. Zn and Mg are dissolved in the Al-matrix and soft inclusions, and they do not form their phases or concentration zones, which was noted by [1]. The EDS point analysis of the Al-7.6Sn alloy (Figure 5a,b) confirms the presence of the following components: Al-solid solution with copper, zinc, and silicon (Point 1), the soft phase inclusions based on tin (Sn-rich phase) (Point 2), soft phase inclusions with Sn and Pb (Sn-Pb and Pb-Sn phases) (Points 3,4), and solid-phase inclusions Al_2_Cu (*θ*-phase) (Points 5,6). The detected phases’ chemical compositions and corresponding EDS spectrums are presented in Figure S1. The formed phases, Al-solid solution, Sn-and Pb-rich phases (Sn-rich, and Pb-Sn phases) and Al_2_Cu (*θ*), were confirmed by the XRD analysis (Figure 6). The soft structural inclusions are located on the dendritic cell boundaries and exhibit a globular and elongated shape (Figure 4a). The globular soft inclusions’ size ranged from 3 to 16 μm, the elongated inclusions’ thickness ranged from 4 to 9 μm, and length ranged from 12 to 38 μm. The distribution of Sn- and Pb-rich phases in a single soft inclusion is depicted in Figure 5a,b. Solid inclusions of the Al_2_Cu phase are primarily located along the dendritic cell boundaries and have an elongated shape with a length ranging from 5 to 45 μm and a thickness from 2 to 10 μm.

The Al-6.4Sn and Al-5.7Sn alloys exhibit a similar microstructure to Al-7.6Sn alloy: a dendritic cell structure with a size ranging from 15–115 μm for Al-6.4Sn alloy and 10–75 μm for Al-5.7Sn alloy (Figure 4b,c). The element distribution maps (Figure 4e,f) also indicate the presence of the Al-solid solution, the soft structural inclusions of Sn-and Pb-rich phases (Sn-rich, Sn-Pb, and Pb-Sn phases), the solid phases inclusions of Al and Cu-rich phase (Al_2_Cu) and a small fraction of the (Si) phase. The presence of these phases was confirmed by XRD-analysis (Figure 6). The soft and solid phase inclusions have a shape and distribution similar to the Al-7.6Sn alloy. For both alloys, the predominant structure of the soft inclusions is the globular shape ranging in size from 3 to 15 μm. In addition, there are a small number of elongated inclusions with a thickness of 2–4 μm and 3–5 μm and a length ranging from 9 to 41 μm and from 10 to 18 μm for the Al-6.4Sn alloy and the Al-5.7Sn alloy, respectively. The solid inclusions of the Al_2_Cu phase are mainly located along the dendritic cell boundaries and have an elongated shape (the thickness ranged from 2 to 5 μm for the Al-6.4Sn alloy and from 2 to 4 μm for the Al-5.7 alloy, respectively). The length of the solid phases usually does not exceed 50 μm for the Al-6.4Sn alloy and 38 μm for the Al-5.7 alloy. The EDS point analysis of the investigated Al-6.4Sn and Al-5.7Sn alloys (Figure 5c–f) also indicates the presence of the structural components: the Al-solid solution with copper, zinc, and silicon, soft phase inclusions with Sn and Pb (Sn-rich, Sn-Pb, Pb-Sn phases), and solid inclusions of Al_2_Cu phase. The detected phases’ chemical compositions and corresponding EDS-spectrums of the Al-6.4Sn and Al-5.7Sn alloys are presented in Figures S2 and S3.

### 3.2. Mechanical Characterization

Table 3 illustrates the mechanical properties of the selected alloys coded as Al-7.6Sn, Al-6.4Sn, and Al-5.7Sn after heat treatment at 250 °C for 3 h and cooling to room temperature with the furnace. The composition of the studied alloys has a substantial impact on mechanical characteristics. (Table 3). The alloy Al-7.6Sn exhibits good mechanical properties, with σ_u_ = 136MPa, δ = 4.7% and 50 HB due to the combined impact of the complex alloying of the alloy. Table 4 summarizes the mechanical properties of the investigated alloys that exhibit the maximum values and the best combinations of the mechanical properties. Generally, the maximum ultimate tensile strength was 197 MPa obtained for the alloy with the chemical composition shown in Table 4, first row. The maximum ultimate tensile strength was reported in alloys with high concentrations of Ni and Cu elements. The maximum HB was 77HB obtained for the alloy with the chemical composition shown in Table 4, second row. From the third to the eighth row of Table 4, the optimal chemical composition combination for good mechanical properties is tabulated. The alloy with the highest concentration of Sn, 1% Pb, and a relatively low value of Cu had the highest elongation to failure (Table 4, third row).

### 3.3. Artificial Neural Network Model

**Figure 7:**
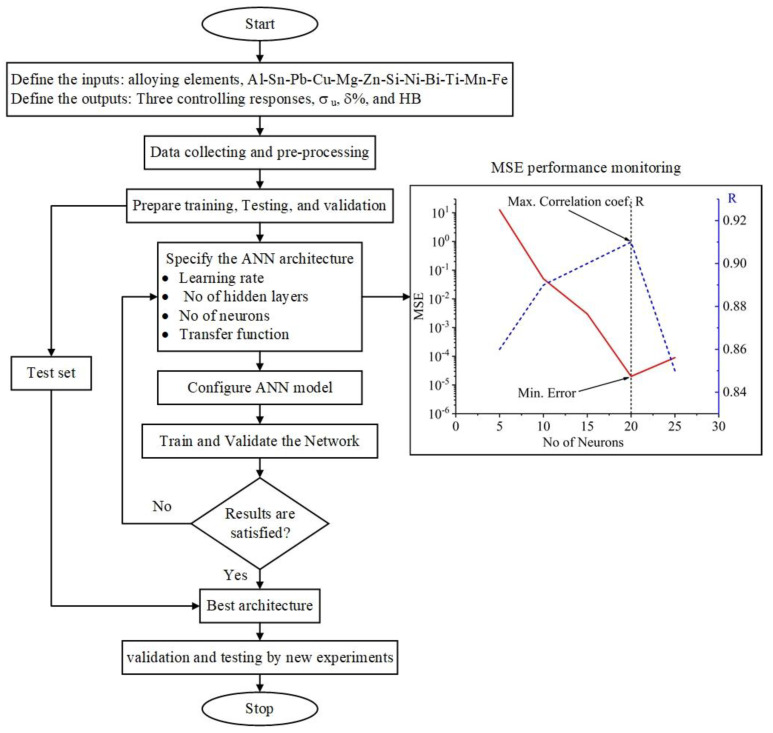
Flow chart of the constructed model and the impact of neurons on the MSE and correlation coefficient.

**Figure 8:**
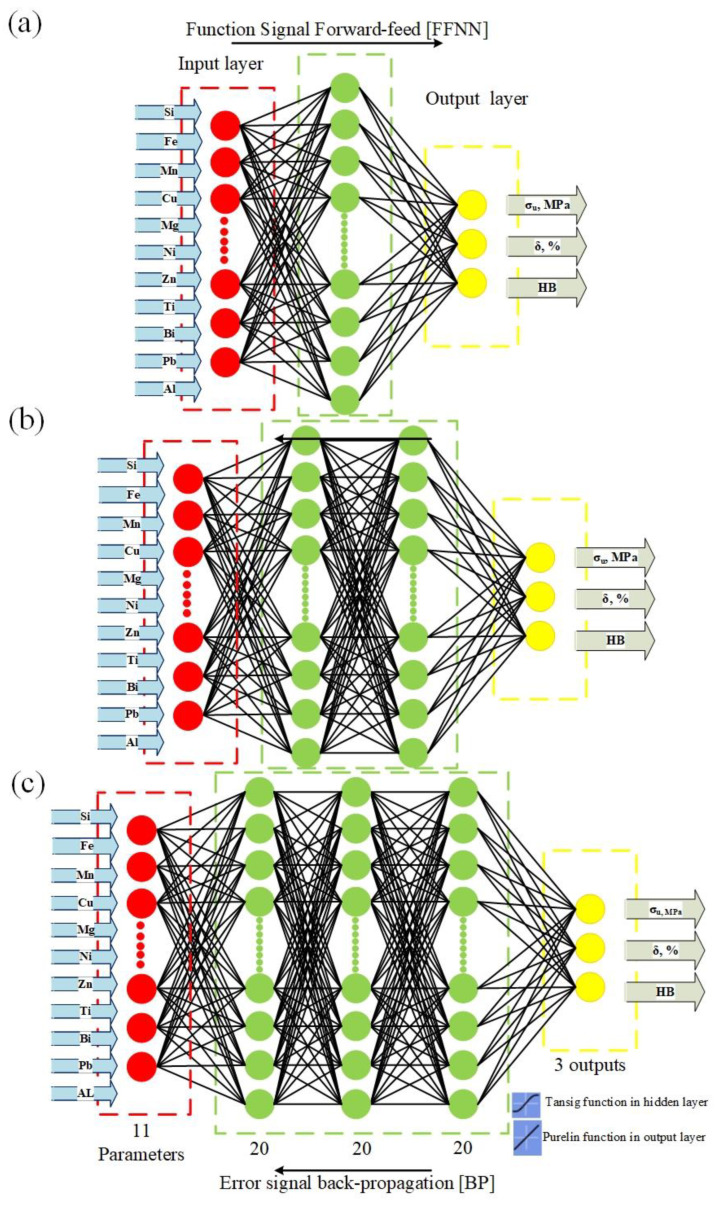
Schematic representation of the utilized ANN architecture: (**a**) one-hidden layer, (**b**) two hidden layers, and (**c**) three hidden layers.

In the present work, the chemical compositions of the added elements (Fe, Si, Mn, Mg, Cu, Ni, Ti, Zn, Bi, Pb, and Sn) of the investigated alloys were selected to be the inputs, and the tensile strength (MPa), elongation to failure (%), and HB were set as the outputs control responses. There were numerous trials, including attempting one, two, and three concealed layers. In addition, various numbers of neurons were tested in each hidden layer. It is well known that the influence of the number of neurons in hidden layers on the output of a network is complex. If the design of the model is overly simplistic, the trained network will not be able to correctly learn the process and determine the relationship between input and output variables. Alternatively, it may fail to align during training, or the training data may be overfitted. Consequently, various network structures in the hidden layer with varying numbers of neurons were investigated. Figure 7 shows the flow chart of the implemented ANN model. The trial-and-errors strategy was launched with one neuron in the hidden layer and progressed with additional neurons to determine the optimal number of neurons. Figure 6 demonstrates how the number of neurons in one hidden layer affects the proposed network’s effectiveness, tested using mean square error (MSE) and correlation coefficient (R) indicators. The MSE was lowest when the number of neurons was 20, corresponding to the maximum linear correlation coefficient (R) value between the experimental and predicted data. Figure 8 schematically illustrates the one (Figure 8a), two (Figure 8b), and three (Figure 8c) hidden ANN architectures, respectively. Prior to training the network, the input and target variables were unified within the range of 0 to 1 to achieve a functional form for the network to read and improve the efficiency of neural network training. Equation (4) is the most commonly used formula for unification.

(4) $$I^{\prime }=\frac{I-0.95I_{min}}{1.05I_{max}-\;0.95I_{min}}\;$$

where

*I* is the experimental data or input data,

*I_max_* and *I_min_* are the max. and the min values in experimental data,

*I’* is the unified value corresponding to *I*.

Training refers to the process of refining network predictions to match experimental data. The network architecture, including the transfer function, training functions, and training methods, must be selected based on the availability of data and the consistency of the output. In this investigation, the tansigmoid and purelinear transfer functions were utilized. The trained network must then be tested to ensure its dependability and precision. The neural network can be trained using various learning algorithms, with the BP algorithm being one of the most common. The weights are modified in an ANN with a BP algorithm, and the changes are saved as knowledge.

This work utilized three different ANN architectures with one, two, and three hidden layers to determine the ideal network’s accurate prediction. For all networks, 11 input neurons (values of Si, Fe, Mn, Cu, Mg, Ni, Zn, Tic, Bi, Pb, and Sn), the hidden layers with 20 neurons, and three output neurons (tensile strength (MPa), elongation to failure (%), and hardness (HB)) were used with ‘tansigmoid’ transfer function for hidden layers and ‘purelinear’ transfer function for outputs. For the network training, a feed-forward backpropagation algorithm is chosen. The used parameters for network training are listed in Table 5. The study was conducted using the MATLAB 2015b software’s neural network toolbox. After approximately 80,000 epochs, the network training was stopped when the goal was achieved and stable. A complete run through a sequence of input-output pairs is an epoch during network training.

**Figure 9:**
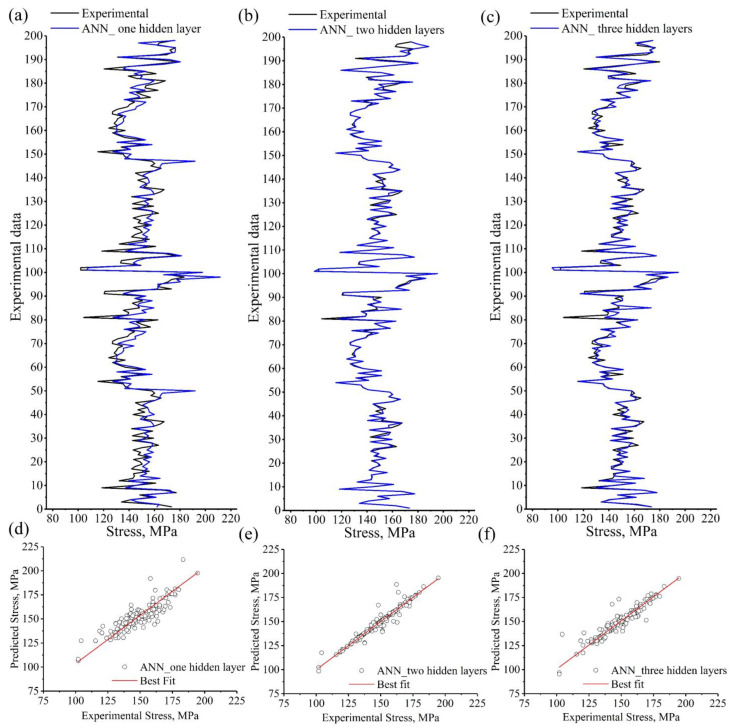
Experimental stress compared to the predicted stress of the ANN model with (**a**,**d**) one layer, (**b**,**e**) two hidden layers, and (**c**,**f**) three hidden layers.

Figure 9 depicts a comparison between the experimental stress (black lines) and the ANN model’s predicted stress with one, two, and three hidden layers and 20 neurons for each hidden layer (blue lines). Table 6 represents the comparison indicators R, RMSE, and AARE (%) between the predicted and experimental stress for the different numbers of the hidden layers. The ANN model with two hidden layers exhibited the strongest correlation between the predicted and experimental stress (Figure 9b,e) compared to the other ANN models (Figure 9a,c,d,f). Using a network with two hidden layers yielded the highest R and lowest RMSE and AARE (%) (Table 6). The ANN model with one hidden layer exhibited undesirable correlation coefficients and error levels between the experimental and predicted stress. Based on Figure 9 and Table 6, the most efficient network contained two hidden layers.

**Figure 10:**
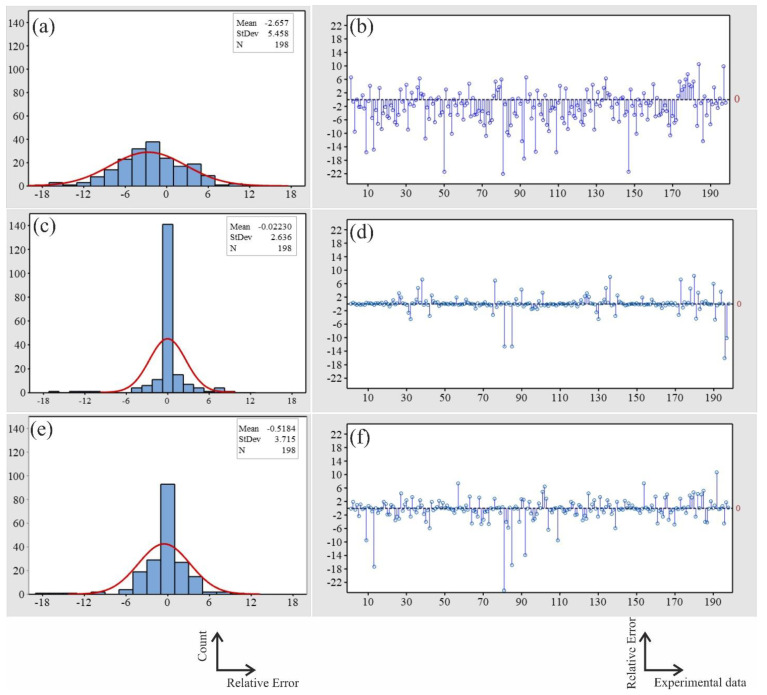
The error histograms and the error plots for the predicted stress of the ANN model with (**a**,**d**) one layer, (**b**,**e**) two hidden layers, and (**c**,**f**) three hidden layers.

Figure 10 shows the relative error histograms and relative error plots of the predicted stress using one, two, and three hidden layers. The relative errors of the predicted stress obtained by two hidden layers were distributed about zero with the lowest standard deviation (SD) of 2.636 (Figure 10c). The distribution of the relative errors of the predicted stress obtained by the one hidden layer was the widest, with an SD of 5.458 (Figure 10a). In comparison, relative errors of the predicted stress obtained by the three hidden layers (Figure 10e) were narrowly distributed compared with those of one layer and wider than those of two layers. The relative error of the predicted stress using two hidden layers ANN model was lower than that of the other used models, namely, one- and three-layers ANN models (Figure 10b,d,f). 

**Figure 11:**
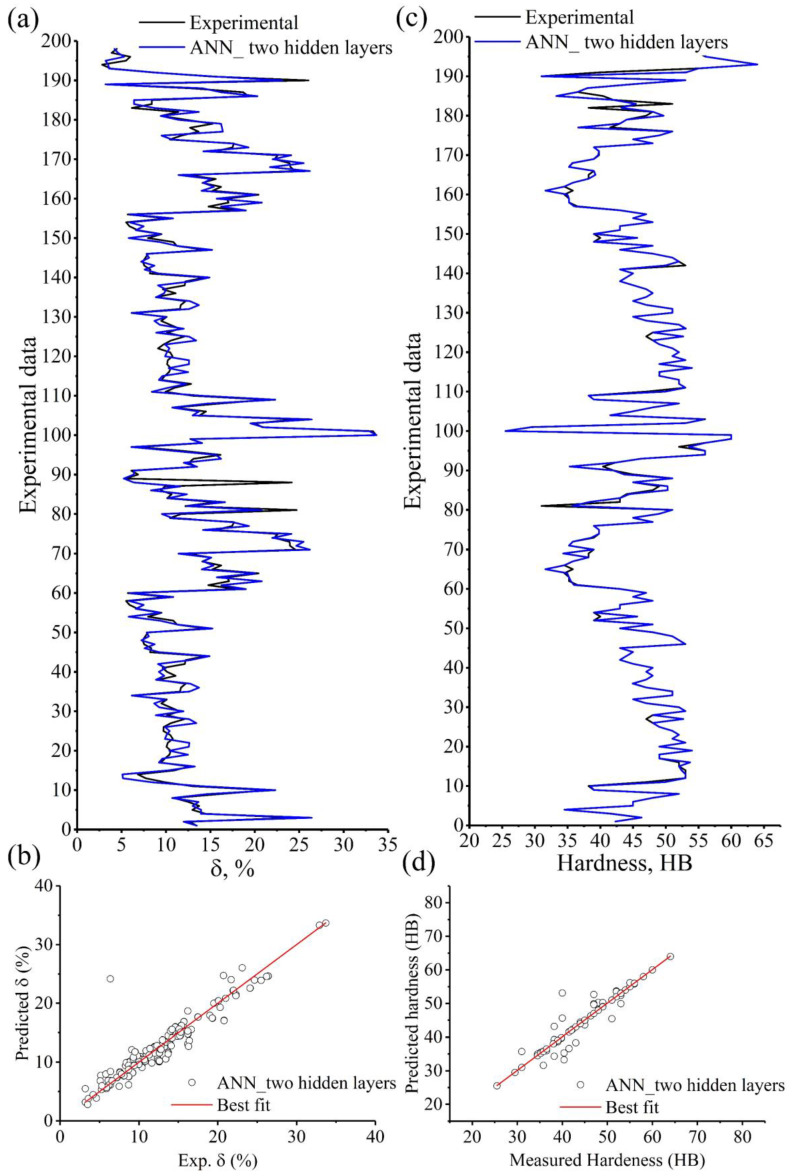
Experimental data compared to the predicted data of the ANN model with two hidden layers (**a**,**b**) elongation to failure, %, and (**c**,**d**) hardness, HB.

Since the ANN model with two hidden layers has proven its efficiency in predicting the stress, it was also used for predicting the elongation to failure (%) and the hardness (HB). Figure 11 illustrates a comparison between the experimental (black lines) and the predicted elongation to failure (%) and hardness (HB) of the ANN model with two hidden layers (blue lines). Table 7 depicts the comparison indicators R, RMSE, and AARE (%) between the experimental and predicted elongation to failure (%) and hardness (HB) using the ANN model with three hidden layers. 

Figure 11 and Table 7 prove the high efficiency of the used ANN model in predicting the elongation to failure (%) and hardness (HB). A stronger agreement was observed between the predicted and experimental hardness (HB) via two hidden layers network (Figure 11a,b) than that for elongation to failure (%) (Figure 11c,d). The all-comparative indicators, R, RMSE, and AARE (%) in the case of hardness (HB), are better than those of the elongation to failure (%), Table 7. Despite that, it is possible to efficiently use the constructed ANN model to predict the elongation to failure (%).

#### Model Verification

Four different alloys were cast and tested to evaluate and verify the precision of the two hidden layers proposed network. Their properties (stress, elongation %, hardness HB) were then predicted. The chemical composition of the casted alloys is tabulated in Table 8. 

**Figure 12:**
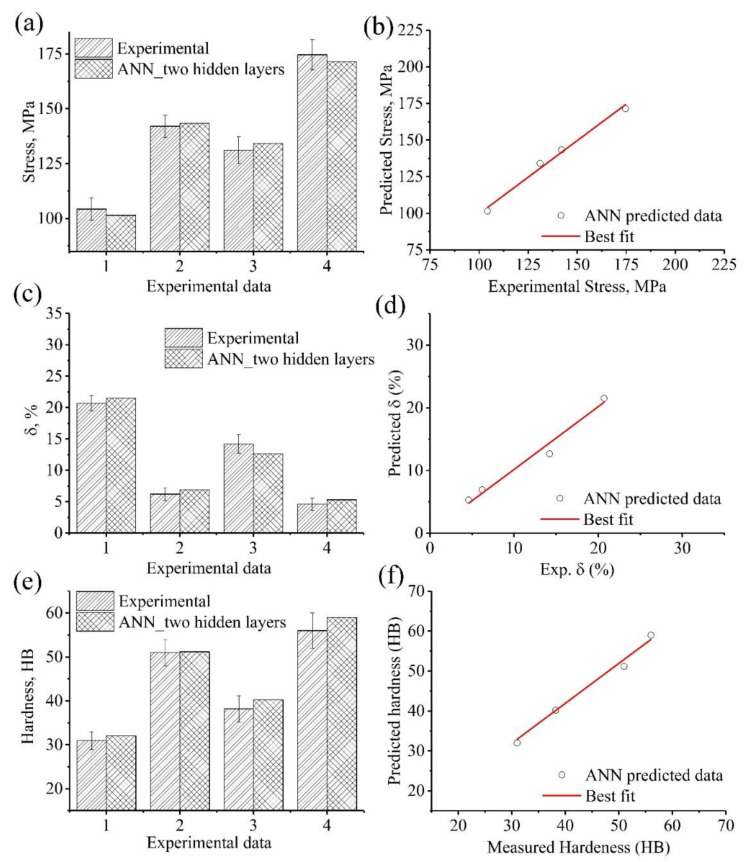
Experimental data compared to the predicted data of the verifications alloys: (**a**,**b**) stress, (**c**,**d**) elongation to failure, %, and (**e**,**f**) hardness, HB.

Figure 12 shows a comparison among the predicted and the experimental stress, elongation to failure (%), and hardness (HB) values for the four alloys (Table 8). Table 9 represents the comparison indicators R, RMSE, and AARE (%) between the experimental and predicted data. The properties of stress, elongation to failure %, and hardness (HB) of the newly prepared four alloys were accurately predicted with a low level of error. The experimental and predicted characteristics were found to be in excellent agreement, as shown in Figure 12. It is observed that the predicted values are located within the interval of the experimental values for all controlled parameters, stress, elongation to failure, and hardness, demonstrating the excellent predictive power of the constructed model. 

The indicator R for the stress was closer to that for the elongation and hardness; it was around 0.97–0.98. In addition, there was an insignificant difference between RMSE for all properties; however, elongation to failure had the lowest value. The predicted properties of stress, elongation to failure %, and hardness (HB), showed a significant difference in AARE %. The largest AARE was obtained for elongation to failure, the highest at 10%, but this is still within the acceptable error range. The prediction of stress yielded the lowest value (1.9%).

## 4. Discussion

The solid solution of Si, Zn, Cu, and Mg in the Al-based solid solution as well as the microstructure analysis of as-cast samples, reveals the presence of Al_2_Cu solid inclusions and soft inclusions based on Sn and Pb (Figure 4 and Figure 5). Zn and Mg are incorporated into the Al-based solid solution and soft inclusions and do not form their own phases [1]. The investigated alloys’ characteristics are compared to those of cast Br.Sn4Zn4Pb17 bronze, which is extensively utilized as a bearing material [15,46]. The Br.Sn4Zn4Pb17 bearing bronze has an ultimate tensile strength of 148MPa, elongation to failure of 8.8%, and hardness of 65 HB [15]. The experimental aluminum alloys (Table 7) exhibit higher strength than those of Br.Sn4Zn4Pb17 bronze. The hardness and elongation to failure vary around those of the Br.Sn4Zn4Pb17 bronze. Alloys 1 and 5 in Table 7 have the best mechanical characteristics of all the aluminum alloys; they are stronger than Br.Sn4Zn4Pb17 bronze and have more plasticity but are characterized by lower hardness. Alloy 3 (Table 7) exhibits larger strength, higher plasticity, and greater hardness than Br.Sn4Zn4Pb17 bronze. 

The mechanical properties, ultimate tensile strength, elongation to failure, and hardness were modeled and predicted using an ANN model. It is well known that the prediction accuracy of the ANN models is significantly affected by the number of hidden layers and the number of neurons in the hidden layers [47,48,49]. The BPANN may not accurately predict when the number of hidden layer neurons is insufficient. However, the more hidden layer neurons there are, the longer the training process will take. A sufficient number of hidden layer neurons should be used to compromise prediction accuracy and training duration. In this work, the minimal MSE was obtained when using two BP network hidden layers with 20 neurons (Figure 7 and Figure 9). It was observed that the relative error of the predicted stress using a two hidden layer ANN model was lower than that of one- and three-layer ANN models. The distribution of the relative errors of the predicted stress about the zero obtained by the two hidden layers was narrower than that of one- and three-layer ANN models. Since the ANN model with two hidden layers has proven its efficiency in predicting the stress, it was also used for predicting the elongation to failure (%) and the hardness (HB). 

In addition, the constructed model with two hidden layers was evaluated and assessed via new experiments. New four alloys were cast and annealed, similar to the modeled alloy, and their mechanical properties: stress, elongation to failure %, and hardness HB, were characterized. The mechanical properties of the new four alloys were predicted by the constructed model to assess the predictability of the BP ANN model. As it showed, the constructed model demonstrated high efficiency in predicting the mechanical properties of these alloys with minimum errors (Table 9 and Figure 12). The correlation coefficient R between the experimental and the predicted mechanical properties of the new four alloys was greater than 0.97. An ANN with 40 hidden neurons was developed by Jimenez-Martinez et al. [50] to determine the chemical constituents of a new aluminum alloy based on the criteria of having a high fatigue strength. The constructed ANN achieved a high level of correlation with an average of 99%. Compared to Jimenez-Martinez et al., our constructed model with 20 neurons in two hidden layers achieved a lower error rate. Fu et al. [51] developed an ANN model with three layers with 12 neurons to model the mechanical properties and microstructure of heat-treated Mg-4.2Zn-1.7RE-Ca-Sr Alloys. In the present study, the inputs consisted of 11 variables, including the contents of the alloying elements, while in Fu et al.’s work, the inputs were Ca and Sr contents and different heat treatment conditions, aging time, and aging temperature. However, the large number of inputs in the present work demonstrated high accuracy in predicting the responses compared with [50,51]. Even though the constructed ANN model accurately predicts the mechanical properties of the designed set of alloys, additional effort is necessary to establish a correlation between the tribological behavior and the mechanical properties of these alloys. Therefore, genetic algorithms will be used in future work to optimize the alloying elements for an excellent combination of tribological behavior and the mechanical properties of these alloys, simultaneously. Consequently, in future research, we will determine the optimal chemical composition to simultaneously achieve maximum wear resistance and mechanical properties.

## 5. Conclusions

The microstructure and mechanical properties of the anti-friction aluminum-based alloys with different concentrations of Sn, Pb, Cu, Mg, Zn, Si, Ni, Bi, Ti, Mn, and Fe elements were investigated. As a result, one hundred ninety-eight alloys were cast from different combinations of the added elements to optimize the chemical composition of these alloys for good mechanical properties and their high wear resistance. The set of experimental data was used to construct an ANN predictive model for predicting their mechanical properties. From the results, we can conclude that:

- The microstructure analysis confirmed the formation of the soft phase inclusions based on tin (Sn-rich phase), soft phase inclusions with Sn and Pb (Sn-Pb, Pb-Sn phases), and solid-phase inclusions of the Al_2_Cu phase.
- The formed phases have a significant impact on the mechanical properties of these alloys. The maximum values of the ultimate tensile strength, HB, and elongation to failure were 197 ± 7 MPa, 77 ± 4, and 20.3 ± 1.0 %, respectively. The obtained levels of mechanical properties are acceptable, besides the excellent tribological properties of these alloys.
- The optimization of the ANN architecture showed that utilizing a network with two hidden layers and 20 neurons for each layer provided a minimal level of errors. For all the modeled mechanical properties, the obtained level in comparative indicators: R ≥ 0.90, RMSE ≤ 3.5, AARE < 8.0%, indicated the constructed model’s high quality.
- The predictability accuracy of the constructed ANN model was evaluated by comparing the simulated data generated by the ANN model to new, unmodeled experimental data. The results demonstrated a high degree of concordance between the predicted and newly acquired data with low error rates. All predicted mechanical properties have comparative indicators of R ≥ 0.97, RMSE = 1–2.65, and AARE < 10%.

In future research, the relationship between the controlled responses (tribological behavior and mechanical properties) will be determined, along with constructing a model based on a genetic algorithm for optimizing the chemical composition for the maximum mechanical properties simultaneously with excellent tribological behavior.

## Figures and Tables

**Table 1:** The chemical composition of the added alloying elements to Al-based anti-friction alloys.

Element	Composition, wt.%		Element	Composition, wt.%	
	From	To		From	To
Sn	4.63	13.77	Pb	0	3.76
Cu	0.45	9.63	Mg	0	0.36
Zn	0	1.44	Si	0.01	5.54
Ni	0	4.15	Ti	0	0.14
Mn	0	0.14	Bi	0	1.04
Fe	0	0.43	Al	Balance	

**Table 2:** The chemical compositions (wt.%) of the investigated alloys.

Alloy	Sn	Pb	Cu	Si	Zn	Mg	Ni	Ti	Mn	Fe	Bi	Al
Al-7.6Sn	7.6	3.3	4.0	1.0	0.5	0.07	0.05	0.06	0.020	0.12	0	Balance
Al-6.4Sn	6.4	2.9	4.1	0.9	1.9	1.4	0.01	0.14	0.001	0.08	0	Balance
Al-5.7Sn	5.7	2.7	4.1	1.5	2.3	1.5	0.14	0.03	0.004	0.14	0	Balance

**Table 3:** Mechanical characteristics of the selected alloys.

Alloy	Ultimate Tensile Strength (σ_u_), MPa	Brinell Hardness (HB)	Elongation (δ), %
Al-7.6Sn	163 ± 7	50 ± 2	4.7 ± 0.5
Al-6.4Sn	136 ± 4	55 ± 3	2.3 ± 0.3
Al-5.7Sn	140 ± 6	55 ± 2	2.1 ± 0.3

**Table 4:** Chemical composition combinations corresponding to maximum and good mechanical properties.

Concentration, wt.%													Mechanical Properties		
1	Si	Fe	Cu	Mn	Mg	Ni	Zn	Ti	Bi	Pb	Sn	Al	σ_u_, MPa	HB	δ, %
2	0.25	0.33	3.61	0	0	4.18	0.01	0.04	0	0	0.36	Balance	197 ± 7	55 ± 3	12.7 ± 0.7
3	0.42	0.25	2.7	0	0	3.7	0.02	0.08	0	0	0.26	Balance	177 ± 6	77 ± 4	17.9 ± 0.8
4	0.14	0.02	1.84	0	0	0	0.34	0.03	0	1.02	7.28	Balance	140 ± 5	37 ± 2	20.3 ± 1.0
5	0.27	0.04	4.54	0	0	0	0.79	0.03	0	2.48	7.21	Balance	180 ± 7	45 ± 2	14.1 ± 0.8
6	0.42	0.06	8.44	0	0	0	1.44	0.04	0	2.23	6.22	Balance	160 ± 6	53 ± 3	3.2 ± 0.4
7	3.85	0.32	1.79	0.09	0.02	0.05	0.48	0.06	0	2.33	7.47	Balance	164 ± 7	55 ± 3	5.3 ± 0.5
8	1.01	0.03	4.49	0	0	0	1	0.03	0	2.1	6.66	Balance	173 ± 7	47 ± 2	8.9 ± 0.9

**Table 5:** Training parameters used in the current work.

Parameter	Contents
Network	BP
Performance function	MSE
Training function	TrainLM
Goal	1 × 10^−7^
Training epoch	80,000
The transfer of the function output layer	Purelin
The transfer function of hidden layers	Tansig.

**Table 6:** The R, RMSE, and AARE (%) of experimental and predicted stress for different numbers of hidden layers.

	One Hidden Layer	Two Hidden Layers	Three Hidden Layers
R	0.72	0.94	0.88
RMSE	7.8	3.5	4.8
AARE, %	3.7	1.0	1.8

**Table 7:** The R, RMSE, and AARE (%) of experimental and predicted elongation to failure (%) and hardness (HB) using two hidden layers network.

	Elongation	Hardness
R	0.90	0.93
RMSE	1.67	1.59
AARE (%)	8	1.1

**Table 8:** Chemical composition of the verification alloys.

Alloy/Elements	Si	Fe	Cu	Mn	Mg	Ni	Zn	Ti	Bi	Pb	Sn	Al
Alloy 1	0.616	0	0.56	0.001	0	0	0.129	0.0301	0	1.95	12.15	Balance
Alloy 2	0.058	0.222	1.69	0.08	0	2.31	0.135	0.046	0.686	2.8	10.53	Balance
Alloy 3	0.741	0.034	0.993	0	0	0.972	0.156	0.05	0.375	1.83	10.14	Balance
Alloy 4	5.24	0.323	1.46	0.144	0.333	0.471	0.351	0.065	0	2.41	7.25	Balance

**Table 9:** The R, RMSE, and AARE (%) of experimental and predicted data of the verification alloys.

Properties	R	RMSE	AARE, %
Stress, MPa	0.98	2.65	1.9
Elongation, δ, %	0.97	1.0	10
Hardness, HB	0.97	2.5	4

## Data Availability

The data presented in this study are available on request from the corresponding author.

## Supplementary Materials

The following supporting information can be downloaded at: https://www.mdpi.com/article/10.3390/ma15238394/s1, Figure S1: SEM-EDS spectrums taken from (a) the aluminium-based solid solution (point 1), the Sn-rich phase (point 2), the Pb-Sn phase (point 3), the Sn-Pb phase (point 4) and the Al2Cu phases (points 5 and 6) in the Al-7.6Sn alloy. Figure S2: SEM-EDS spectrums taken from (a) the aluminium-based solid solution (point 1), the Sn-rich phase (point 2), the Pb-Sn phase (point 3), the Sn-Pb phase (point 4) and the Al2Cu phases (points 5 and 6) in the Al-6.4Sn alloy. Figure S3: SEM-EDS spectrums taken from (a) the aluminium-based solid solution (point 1), the Sn-rich phase (point 2), the Pb-Sn phase (point 3), the Sn-Pb phase (point 4) and the Al2Cu phases (points 5 and 6) in the Al-5.7Sn alloy.

## Nomenclature

ANN	Artificial neural network
ML	Machine learning
BP	Backpropagation
MSE	Mean square error
I	Experimental data or input data
I_max_	The maximum value in experimental data
I_min_	The maximum value in experimental data
I’	The unified value corresponding to Y
BP	Backpropagation algorithm
R	Correlation coefficient
RMSE	Root mean square error
AARE	Average actual relative error
*E_i_,*	Experimental values
*P_i_*	Predicted values
$\bar{E}$	Average of the experimental values
$\bar{P}$	Average of the predicted values
*N*	Number of the casted alloys
σ_u_	Ultimate tensile strength
HB	Brinell hardness
δ	Elongation to failure
