# Supplementary material for: Bearing Aluminum-Based Alloys: Microstructure, Mechanical Characterizations, and Experiment-Based Modeling Approach
Source: Materials (Basel). 2022 Nov 25;15(23):8394. doi: 10.3390/ma15238394 (PMC9735459; doi:10.3390/ma15238394)
Supplement: Supplementary file 1 [file materials-15-08394-s001.zip › materials-1978791-supplementary.pdf]

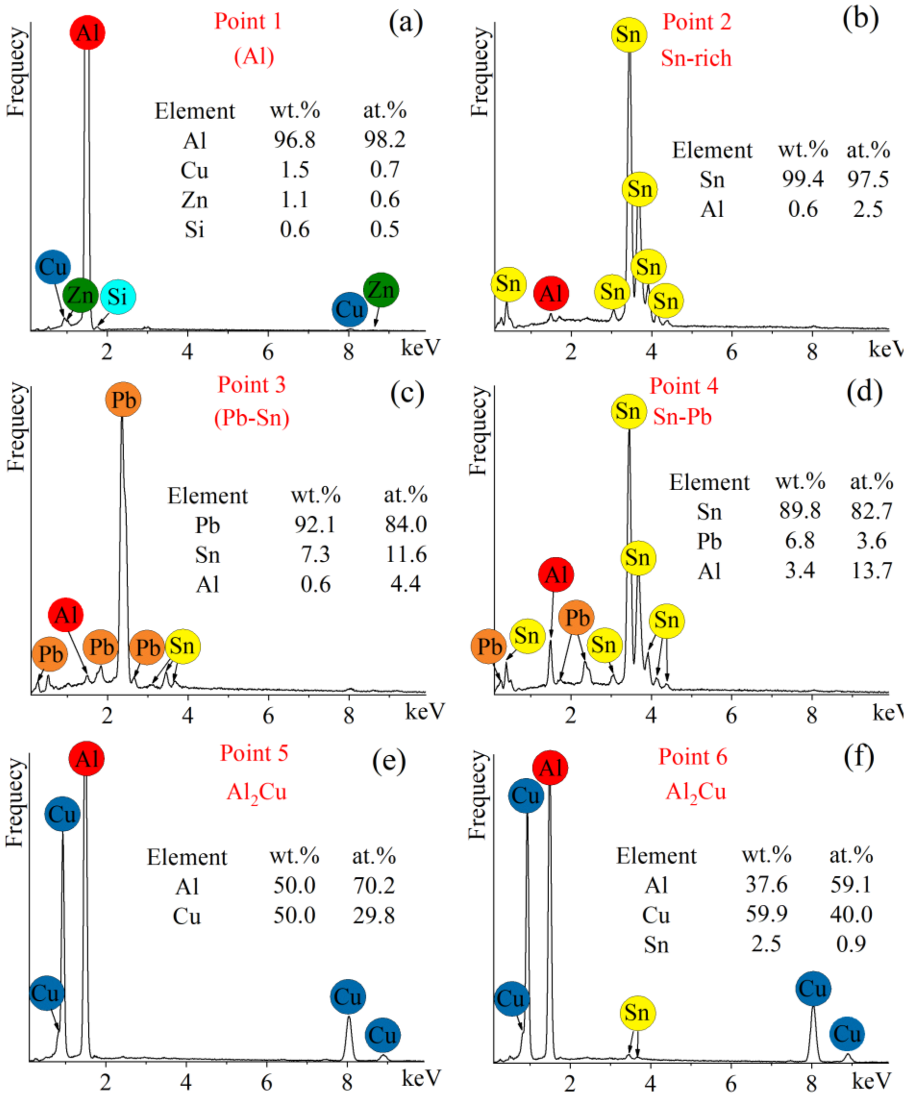

Figure S1. SEM-EDS spectrums taken from (a) the aluminium-based solid solution (point 1), the Sn-rich phase (point 2), the Pb-Sn phase (point 3), the Sn-Pb phase (point 4) and the Al_2Cu phases (points 5 and 6) in the Al-7.6Sn alloy.

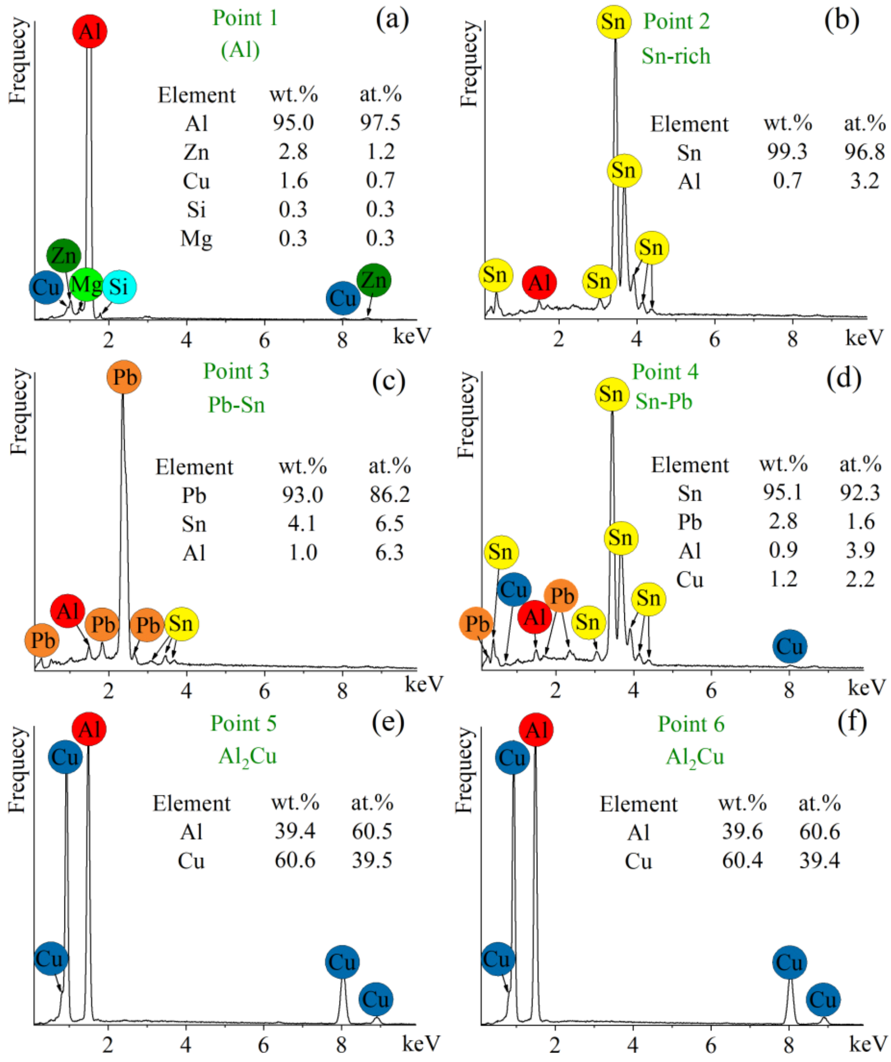

Figure S2. SEM-EDS spectrums taken from (a) the aluminium-based solid solution (point 1), the Sn-rich phase (point 2), the Pb-Sn phase (point 3), the Sn-Pb phase (point 4) and the Al_2Cu phases (points 5 and 6) in the Al-6.4Sn alloy.

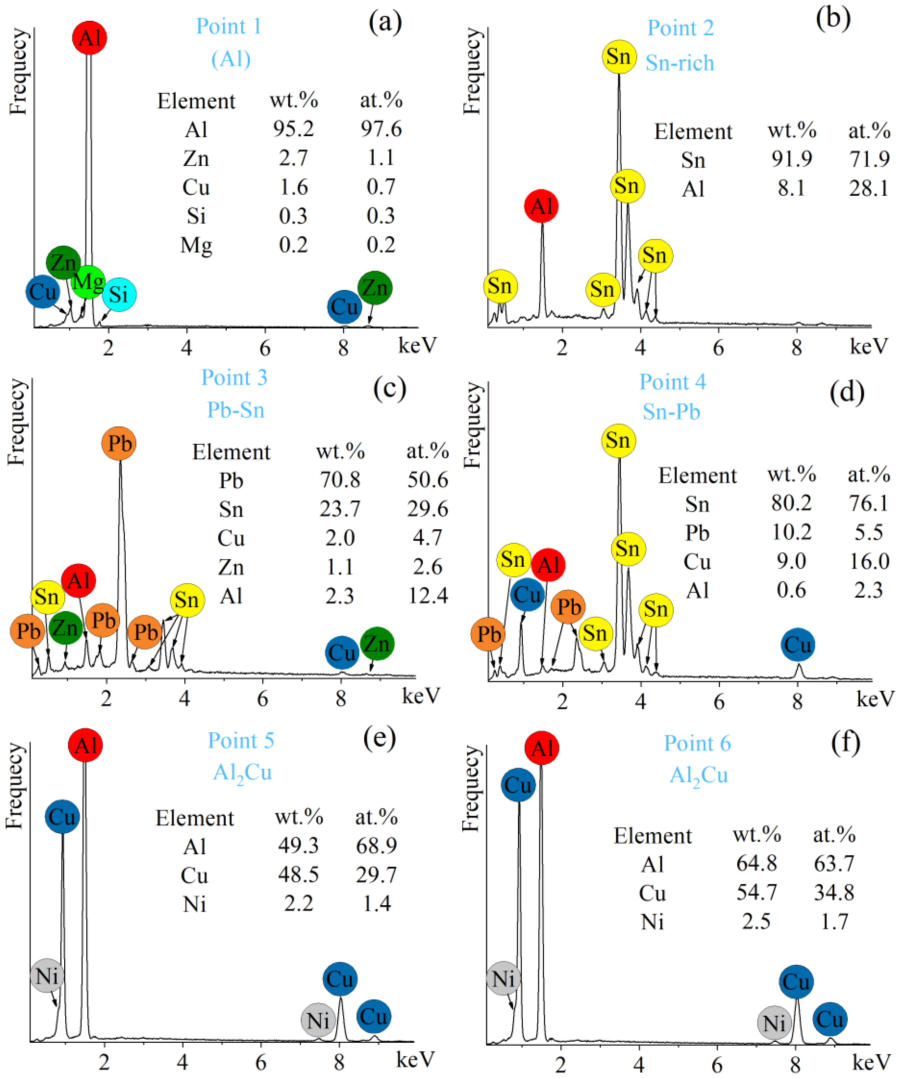

Figure S3. SEM-EDS spectrums taken from (a) the aluminium-based solid solution (point 1), the Sn-rich phase (point 2), the Pb-Sn phase (point 3), the Sn-Pb phase (point 4) and the Al_2Cu phases (points 5 and 6) in the Al-5.7Sn alloy.
